# Editorial: Psychological and neurobiological mechanisms of time perception and temporal information processing: insight from novel technical approaches

**DOI:** 10.3389/fnbeh.2023.1208794

**Published:** 2023-06-09

**Authors:** Fuat Balcı, Koji Toda

**Affiliations:** ^1^Department of Biological Sciences, University of Manitoba, Winnipeg, MB, Canada; ^2^Department of Psychology, Keio University, Tokyo, Japan

**Keywords:** interval timing, computational modeling, time perception, magnitude representations, virtual reality

Subjective time is an integral part of our experience and cognition. Decades-long research has suggested that animals, including human has a mechanism of the internal clock/stopwatch (Gibbon et al., 1984; Buhusi and Meck, 2005; Merchant et al., 2013; Paton and Buonomano, 2018). The similarities between the psychophysical properties of timing behavior observed in human and non-human animals suggest that the mechanism of timing and time perception is an evolutionarily well-preserved neurobiological mechanism. Based on this premise, researchers have made a leap in understanding the internal clock/stopwatch based on the comparative study of interval timing. However, the knowledge of the psychological and neurobiological mechanisms of timing is still far from complete.

This Research Topic focused on recent technical and theoretical developments in the study of interval timing. De Corte et al. has made an excellent conceptual analysis of interval timing models and their implications for neural computing. They classified models of interval timing as ramping vs. population code models and discussed the computational affordances of these theoretical approaches. They showed the importance of understanding the neural mechanisms of temporal scaling and suggested understanding the mechanisms of speed regulation, which enable the model to show temporal scaling as the next research direction.

An empirical gap in the study of interval timing, particularly in non-human animals, is that the related behaviors are studied in a fashion that is isolated from the ecologically relevant setting. In this issue, Henke et al. introduced a new behavioral task in virtual reality that required animals to reproduce the target duration walking along a corridor. Importantly, subjects (i.e., gerbils) could not use spatial cues as a proxy for time and exhibited timing performance that highly resembles human data (i.e., scalar variability, regression to the mean). The use of virtual reality and its coupling with neuroscience methods will surely be one of the principal axes of future behavioral neuroscience research.

Another way of increasing the ecological validity of the findings regarding interval timing behavior is to include those individual-state factors that commonly vary in nature within and between individuals. The motivational state of the animals is one of those factors. Pérez-Calzada and Oscar Zamora showed that delivering reward during the inter-interval interval leads to an evident change in the psychophysical function for temporal bisection, highlighting the need to consider motivation as a relevant factor in the study of interval timing.

The interaction between numerosity and time perception has been studied. A theory of magnitude suggests that a generalized magnitude system in the brain processes magnitudes such as space, time, and numbers. Although numerous behavioral and neurocognitive studies have provided support for a theory of magnitude, the evidence for common magnitude processing is controversial. The final paper in this issue by Shukla and Bapi tests the predictions of a generalized magnitude system in relation to cross-modal interactions. They show that the interactions between different magnitudes (i.e., numerical and temporal) might be mediated by general cognitive mechanisms rather than a common magnitude system.

Briefly, the series of research and review articles that compose this Research Topic covered a wide range of technical and theoretical studies in the study of interval timing. Recent advances in behavioral and neuroscientific techniques of measurement and manipulation, such as computer vision, motion tracking, optogenetics, chemogenetics, *in vivo* calcium imaging, reinforcement learning, and deep learning offer new opportunities for a more comprehensive understanding of mechanisms that underlie interval timing (e.g., Soares et al., 2016; Toda et al., 2017). Implicating these techniques in the conceptual theoretical analysis will be an important step to fully understanding the mechanism of timing and time perception.

## Author contributions

All authors listed have made a substantial, direct, and intellectual contribution to the work and approved it for publication.
